# Mental Health of First Responders in Rural Australia: A Qualitative Exploration of Intersecting Individual, Organisational and Contextual Factors on Psychological Wellbeing

**DOI:** 10.1111/inm.70349

**Published:** 2026-09-08

**Authors:** Rikki Jones, Kylie Rice, Debra Jackson, Kim Usher, Jamie Ranse, Aimee Gayed, Clare Sutton, Lisa Clegg, Andrew Arena

**Affiliations:** ^1^ University of New England Armidale New South Wales Australia; ^2^ Manna Institute University of New England Armidale New South Wales Australia; ^3^ University of Sydney Sydney New South Wales Australia; ^4^ Griffith University South Brisbane Queensland Australia; ^5^ Black Dog Institute University of New South Wales Randwick New South Wales Australia; ^6^ Charles Sturt University Port Macquarie New South Wales Australia

## Abstract

To explore the factors shaping mental health experiences among rural and remote first responders and to examine how organisational and contextual conditions influence psychological wellbeing. First responders are routinely exposed to traumatic events and are at elevated risk of mental health difficulties. In rural and remote settings, geographic isolation, limited workforce capacity, and reduced access to specialised services may compound these risks. However, less is known about how workplace culture and service structures interact with trauma exposure in these contexts. A participatory action research (PAR) approach was used to co‐design this study, using phenomenological qualitative methods through semi‐structured interviews with rural and remote first responders. Data were analysed using reflexive thematic analysis informed by phenomenological principles to explore lived experience. An advisory group contributed to the interpretation and refinement of findings. Participants described cumulative mental health strain arising not only from trauma exposure but also from structural and cultural conditions embedded within rural service environments. Geographic isolation, small team structures, high workload, and limited anonymity intensified emotional labour. Workplace cultures characterised by expectations of stoicism, ‘hero’ narratives, misogyny and gender discrimination, and limited recognition contributed to moral distress and psychological burden. Participants identified significant gaps in mental health service provision, including limited access to trauma‐informed and first responder‐specific supports in rural areas. Mental health challenges among rural first responders are shaped by intersecting individual, organisational, and structural factors. Trauma exposure alone does not account for the complexity of distress experienced in these settings. Interventions must extend beyond individual resilience‐building to include organisational reform, psychologically safe workplace cultures, and improved access to tailored, trauma‐informed mental health services in rural and remote contexts.

## 1 Introduction

Exposure to traumatic events has a profound impact on mental health and repeated or cumulative trauma experiences can increase the risk of developing post‐traumatic stress disorder (PTSD) (Jones, Jackson, Ranse, et al. 2024; Milligan‐Saville et al. 2018). These risks are particularly relevant for first responders, who are often called out to attend potentially traumatic incidents that are severe, unpredictable, complex, and dangerous, involving physical violence, sexual assault, childhood sexual abuse, death and human suffering, that may place the first responders' safety at imminent risk (Jones, Jackson, and Usher 2024; Murray et al. 2020). First responders are defined as those undertaking any job that involves being the first on scene for an incident involving an emergency, accident, or disaster (Jones et al. 2025). For many first responders, these incidents can result in a trauma response and increase the risk of developing PTSD, with reports that more than 14% of first responders meet the clinical threshold (Arena et al. 2025). First responders are also at increased risk of experiencing other mental health issues such as anxiety, psychological distress, depression, panic disorders and suicidal ideation (Jones, Jackson, Ranse, et al. 2024; Lee et al. 2022; Szeto et al. 2019). Research conducted within Australia is consistent with international findings of heightened risk of mental health issues and suicidality among first responders, impacts of cumulative trauma and negative wellbeing outcomes of trauma (Kerswell et al. 2020; Kyron, Rikkers, Bartlett, et al. 2022; Kyron, Rikkers, LaMontagne, et al. 2022; Kyron et al. 2021; Milligan‐Saville et al. 2018; Smith et al. 2022).

For first responders working in regional, rural and remote (RRR) locations, the impact of traumatic experiences at work may be even more pronounced than their urban counterparts due to multifaceted issues specific to these contexts (Jones, Jackson, Ranse, et al. 2024; Jones, Jackson, and Usher 2024). For example, rural and remote locations often have limited staffing and other resources that impact staff safety, first responders respond to people who may be known to them in the rural community and may increase exposure to traumatic incidents, high workload and staff burnout (Jones, Jackson, and Usher 2024). Additionally, there are added challenges in RRR areas with the delivery and access to mental health support, barriers to help‐seeking and concerns around privacy and confidentiality when accessing services (Jones et al. 2025).

Despite this increasing recognition of the issues faced by first responders in RRR areas, research remains limited. A recent scoping review undertaken by Jones, Jackson, Ranse, et al. (2024) reported a dearth of evidence focusing on the specific issues for first responders working in RRR Australia. The scoping review reported several significant gaps in evidence including limited qualitative research exploring lived experience, lack of high‐quality research studies exploring the effectiveness of interventions in RRR populations, limited examination of the specific traumatic incidents experienced in RRR areas and lack of understanding of the key mental health issues for first responders working in RRR areas (Jones et al. 2025). There is an urgency for more research to be undertaken at national and international levels on rural mental health (Roberts et al. 2024). Hence this study aims to uncover first responders' experience of working in RRR Australia, with particular attention to mental health issues and challenges. Specifically, the study aims to:

1. Explore the key mental health issues for first responders working in RRR Australia.
2. Examine the challenges related to mental health in RRR areas and determine whether this is affected by rurality.
3. Explore what initiatives, interventions, and strategies are used to support rural first responders' mental health.

## 2 Methods

### 2.1 Co‐Design

A participatory action research (PAR) approach was used to co‐design this study, which uses phenomenological qualitative methods to explore first responder and health care professional lived and living experience in RRR areas of Australia. A PAR approach promotes equal collaboration among key stakeholders (rural first responders, researchers and end users [health professionals]), working as a team with the researchers to ensure the research is collaborative, gives consumers/participants power to drive meaningful change and ensures priorities of consumers are embedded in research (Cornish et al. 2023). An advisory group (AG) was set up at the commencement of the project to ensure a PAR approach was followed. The AG was recruited through research team networks and key organisations via email and social media posts. A total of 13 RRR first responders (firefighters *n* = 3, State Emergency Service [SES] *n* = 3, paramedic/ambulance *n* = 3, police *n* = 4) and 6 health care clinicians who work with RRR first responders were recruited to the AG. The research project was developed in consultation with the AG to ensure their expertise was guiding the project from development to reporting of the findings. Throughout the project the research team met with the AG bi‐monthly to gain feedback and insights at each stage (including project development, research design/methodology, research aims/questions, interview guide, recruitment, data collection, data analysis, interpreting and reporting of results and dissemination of findings). The PAR approach is well‐aligned with phenomenological methods, which aim to explore, understand and interpret the lived experience of a phenomenon (Matua and Van Der Wal 2015).

### 2.2 Setting and Participants

This study recruited both first responders and health care professionals using the AG networks and members of the AG were eligible to participate in this stage of the research project. Table 1 outlines the inclusion and exclusion criteria for participants in this study. Participants were recruited from across Australia.

**TABLE 1:** Inclusion/exclusion criteria for participation.

Inclusion for first responders First responders (including police, paramedic/ambulance officer, SES, Volunteer Rescue Association [VRA] and fire Fighters/RFS) who have experience working in rural, regional and remote areas of Australia. A minimum of 4 years' experience working in the relevant role. Currently employed or current volunteer or recently retired.
Inclusion for health care professionals Australian professionals/clinicians who have experience working with rural, regional and remote first responders with mental health issues. 4 years' experience working in the relevant role. Experience treating first responders with mental health issues.
Exclusion criteria Does not meet the requirements for first responder or health care professional. Not fluent in written and/or oral English. Under the age of 18.

### 2.3 Recruitment and Ethical Considerations

This study received ethical approval from the university Human Research Ethics Committee (HE24‐066) Recruitment involved strategic advertisement (via emails and flyers) through the advisory group and researchers' networks, using a snowballing technique. All potential participants were required to email the research team to discuss participation, ensure eligibility for the study, to receive the information sheet for participants (ISP) and consent forms. Written consent was returned via email to the lead researcher prior to allocation to one of the focus groups or interview (determined by availability and/or preference); time was organised. A pseudonym was allocated to each participant prior to commencing the audio recording to ensure identifying details were not collected during the process.

### 2.4 Data Collection

Semi‐structured interviews and focus groups were undertaken between May and September 2024. An interview protocol was used to guide both interviews and the focus groups (see Table 2). The interview protocol was developed in consultation with the advisory group and informed by a scoping review undertaken previously by the research team (Jones et al. 2025). The semi‐structured interview protocol enabled the researcher to collect similar data across the different formats (interview/focus groups), while tailoring follow‐up questions to suit each session (DeJonckheere and Vaughn 2019; Nowell et al. 2017).

**TABLE 2:** Interview protocol.

Demographics – What organisation are you affiliated with, are you associated with multiple organisations? If so, which ones. – How long have you been affiliated with these organisations? – Where are you located (what rural community) and what community/communities do you work in/for as a first responder?
What are the key mental health issues for first responders working in rural Australia? Can you outline a situation that would help me understand these issues?
Is the mental health and resilience of first responders affected by rurality? Can you provide some examples?
Do you believe first responders in regional, rural and remote Australia are likely to access mental health care for mental health issues related to their role and if so, where would they seek help?
What initiatives/strategies or resilience traits or abilities have you employed or seen work in real‐world setting to help first responders in rural Australia respond to and better manage their mental health? Are any of these currently available? If so, can you explain where they are available.
What are some examples of responders experiencing challenges or barriers to accessing mental health care services in rural areas?
How does the mental health of first responders' impact their families? Can you tell me a story that help outlines these experiences for family members.
Is there anything else anyone would like to add?

Participants nominated if they wished to attend a focus group or an interview, via zoom or teams and all were led by the first author to ensure consistency. The audio recordings were transcribed using an approved transcription company. Transcripts were reviewed by the researcher (Rikki Jones) to ensure accuracy of the transcription.

### 2.5 Data Analysis

Reflective thematic analysis following Braun and Clarke (2019) six‐step process was employed, informed by phenomenological principles concerned with understanding lived experience (Ranse et al. 2020). These phenomenological principles include remaining oriented to the phenomena being explored, participant narrative providing concrete descriptive accounts of the phenomena and the analysis uncovering the singularity or uniqueness of an experience.

Three researchers (Rikki Jones, 2 research assistance‐ RF, NB) independently analysed the data using this six‐step process; familiarisation with data, code identification, coding of all transcripts, developing connections and links between codes, identifying common themes and writing up results. To support analytical rigour and guard against superficial theme development, we maintained a transparent audit trail of analytic decisions, engaged in ongoing reflexive discussion within the research team and prioritised interpretive depth while remaining grounded in participants' accounts (Watson and Jackson 2026). In keeping with contemporary discussions of descriptive and interpretive phenomenology, we recognised that meaning is shaped through context and analytic engagement with the data (Sundler et al. 2019; Watson 2025).

Accordingly, the analysis aimed to move beyond code listing to develop coherent patterns of shared meaning across the dataset, while retaining closeness to participants' language and context (Watson and Jackson 2026). The lead author and research assistants immersed themselves in the data, reading and rereading transcripts and listening to recordings to ensure familiarity with the data and to identify the initial codes. As part of the PAR approach, a list of the initial codes with examples was presented to the research team and AG for discussion and to ensure interpretation was undertaken through a lived‐experience lens. A coding tree was maintained by the lead researcher throughout the process with changes recorded until a final list of codes was generated under consultation with the research team and the AG. Two research assistants used NVivo to continue coding the data under the guidance of the lead researcher. Once coding was completed, the research team and the AG explored the codes (with examples) to identify connections and meaning between codes, to group codes into subthemes and to group subthemes into themes. The themes, subthemes and codes were then presented to the AG for further refinement and review. This rigorous process was employed to ensure the results were credible, transparent and well‐grounded in the dataset (Braun and Clarke 2019; Nowell et al. 2017) and to interpret the findings through the lived experience lens which fits with the PAR approach of co‐designing research.

## 3 Results

A total of 14 participants were recruited into the study (focus group *x*1 [*n* = 4], two‐person interview × 2 [*n* = 4] and single interviews × 6 [*n* = 6]); 79% of the participants were female (male *n* = 3, female *n* = 11) and 70% identified as a first responder (RRR first responder *n* = 10 or RRR health care professional *n* = 4). Two first responders were medically retired, nine were currently employed in their role, four were volunteers and one was retired (see Table 3), all worked in RRR areas across New South Wales (NSW, *n* = 11), Victoria (VIC, *n* = 1) or Queensland (QLD, *n* = 2). Of the four health care professionals working with first responders, one was a psychologist, one a social worker and two were mental health nurses. A total of 368 min of data was recorded (focus group = 98 min, 2‐person interviews average 32 min, individual interviews average 35 min). Table 3 presents the participants' characteristics. All participants were allocated a pseudonym to ensure they remained de‐identified.

**TABLE 3:** Participant characteristics.

Pseudonym	Gender	Role	Role status	Years of experience	Data collection
First responders					
Simon	Male	Police Fire Service	Retired Volunteer	20–30 years 10–20 years	Interview
Emma	Female	State Emergency Service (SES)	Employed	4–10	Focus Group
Jay	Female	Paramedic	Employed	10–20 years	Interview
Lucas	Male	Police	Retired	10–20 years	Interview
Maise	Female	Fires Service	Volunteer	20–30 years	Focus Group
Ruth	Female	SES Police	Retired Prior Employment	4–10 years 40+ years	Interview
Suzette	Female	Ambulance‐ Chaplain & Paramedic	Employed	10–20 years	Focus Group
Sharn	Female	Rural Fire Service SES Red Cross	Volunteer Volunteer Volunteer	4–10 years	Interview
Tate	Male	Paramedic SES	Employed Volunteer	30–40 years	Interview
Laura	Female	SES PHC Endorsed Nurse Practitioner	Volunteer Employed	10–20 years 4–10 years	Interview
Health professionals					
Dede	Female	Health‐ Mental health nurse	Employed	30–40 years	Focus Group
Karie	Female	Health‐ Mental health nurse	Employed	4–10 years	Interview
Marissa	Female	Health‐ Psychologist	Employed	10–20 years	Interview
Tabitha	Female	Police‐ Mental health Health‐ Mental health social worker	Employed Prior employment	4–10 years 4–10 years	Interview

Data analysis revealed one three main themes, (1) Rural practice involves unique workplace and community challenges that impact mental health, (2) Unique barriers with rural mental health services hinder mental health support and (3) Effective mental health support requires greater cultural sensitivity to rural contexts, these are presented in Table 4. Participant quotes are included for each theme and additional quotes for each theme can be found in the supplementary file (Table S1). The findings reported by health professionals and first responders were consistent across all themes, hence the results have been reported as one and differences between health professional and first responders noted where appropriate.

**TABLE 4:** Theme, subthemes, threads.

Rural practice involves unique workplace and community challenges that impact mental health	
Organisational issues/culture	Misogyny and discrimination
	Cultural load
	Moral injury
	Safe workplaces
	Work pressure and load
	Mental health support
	Low volunteer recognition and promotion
Workforce and professionalism	Demand for professionalism
	Inexperienced workforce
	Expanded work roles
*Rural connections*	Ethical challenges
	Community acceptance
	Hero mentality
	Community expectations
Unique barriers with rural mental health services hinder mental health support	
Challenges with rural services	Complex relationships
	Transient workforce & services
	Disparity between rural and urban
	Support mechanisms
Service Acceptability	Trust and connection
	Consistency
	Understanding the issues
Service Accessibility	Service availability and information
	Distance
	Financial Constraint
	Poor Internet Service
Perceived impacts of identifying MH issues	Burden of Proof of identity
	Perceived repercussion
	Stigma
Effective mental health support requires greater cultural sensitivity to rural contexts	
Empowerment	Community empowerment
	Individual empowerment
	Perceived value
	Strengthening protective factors
Individual strategies to mitigate mental health related issues	Appropriate support for retired personal
	Developing Resilience Skills
	Diet and exercise
	Hobbies & self‐care
Support & interventions	Awareness, education & prevention
	Clinical supervision
	Collaboration
	Debrief
	Mental health treatment

### 3.1 Rural Practice Involves Unique Workplace and Community Challenges That Impact Mental Health

This theme describes the rural workplace and community factors that exacerbate mental health challenges and the impact trauma has on first responders. These included organisational culture, workforce professionalism and rural connections. Table 4 presents the subthemes and threads identified during data analysis.

Organisational factors that add strain on first responders are compounded in rural contexts. Both first responders and health care professionals highlighted how first responder organisations sometimes viewed mental health as a check box activity, when it is rather a work health and safety concern that requires more strategic considerations and structured interventions. In RRR contexts, this organisational attitude is viewed as particularly inadequate, as it inappropriately minimises the added strain on first responders' mental health due to the nature of practising in these areas, including increased professional and social isolation, increased exposure to traumatic incidents and a perception of increased burden due to reduced resources (such as staffing). These factors impacted first responders' desire to remain in the role and often led to burnout which adds additional pressure by further reducing staff in RRR areas.

I feel like the further out you are the more compounded those things become. Because it's up to you or no one else. There isn't—there aren't other services. There aren't other units. There aren't other members available. So, yeah, you have that feeling of burden of responsibility so you can't take your own self‐care seriously. Then that's where the burnout happens. (Emma)

The pressure from organisations on first responders to remain certified and competent placed strain on first responders, which was also exacerbated by working in RRR contexts. Despite the lack of staff, which is increasingly problematic as remoteness increases and first responders reporting they were unable to take time off, they were still expected to maintain a level of competence that was demanding. This was also reported for volunteer first responders who must maintain competency requirements in addition to their full‐time role. With rural and remote areas this was exacerbated with limited trainers available to help maintain competency to ensure staff remained certified. Additionally, in rural and remote areas, first responders had to employ broader skill sets and work outside the normal first responders' scope of practice to meet the demand of the profession.

The rural centres don't have enough trainers to keep up the accreditation so we're travelling people to [metropolitan suburb] to have training. So, yeah, the burden of remaining accredited—if you can't get on your knees to do CPR, we don't need you anymore, you can't be clinical. So if you've had a knee replacement, or all of the sudden you've got something that's happened to your knees and you can't kneel down to do your pump and jump then you're no longer competent. You're no longer required. So, yeah, training does put a lot of burden on whether you can or can't belong, at what level you can and can't belong and can you remain in the organisation. (Suzette)

First responders faced several ethical challenges due to the nature of RRR communities, including issues of privacy and confidentiality. First responder participants reported feeling they had a social responsibility to community members. The complex relationships between first responders and the community were impacted by how well the first responders performed their job, if the first responders were seen to have made a mistake while undertaking their role, the community sometimes lost respect for that profession and this impacted how they engaged with the first responder.

I just think being in remote towns, rural towns as a health professional and emergency service—and you've heard about it or you've seen it happen, once the community loses respect in that profession—we had a police officer in our town and the respect was gone for that person, for something that seemingly was just a small thing. People are allowed to make mistakes and it didn't really hurt any—it's just, that was the attitude of the town. Their responses to the police were different. Some other friends had some random stuff happen to different paramedics in smaller towns and the judgement of the town is a huge thing. (Jay)

Acceptance from the community was important and without respect and acceptance, first responders felt powerless and unable to function in their role. Additionally, rural communities felt a sense of ownership of the first responders, with participants reporting this as both a positive (belonging to the community they serviced) and a negative (the community owned them and they had to meet the communities' expectations) experience. First responders in this sample who did not feel acceptance within the community reported an increased sense of isolation and disconnection, which was exacerbated for first responders not born in the area, whose family and social support were elsewhere.

It's quite a complex thing and community members, particularly in large‐scale events, constantly turning to first responders wanting updates. So, their over‐heightened sense of awareness and social responsibility is increased, as is their sense of impotency as well. Yeah. They're just a few of the things that are particularly unique to remote and rural responders versus people who probably live in larger towns, cities…(Maise)

Another challenge of the complex relationships between rural communities and first responders was the hero worship mentality. First responders often felt like their sense of worth within the community was tied to their role as a first responder and this could be difficult, especially for volunteers, who had to return to their regular day jobs after the community crisis had resolved. Rural communities had an increased expectation of what first responders could and should be able to do and this was seen to have a massive impact on first responders' mental health. First responders reported feeling a sense of guilt if they had to take time off during extended disasters (such as drought and fires). This expectation to always be available to respond impacted time off with family, so even if the first responder was not on call, they felt guilty because they were not there to respond to emergencies. Volunteer first responders also felt they were used to reduce costs and had to juggle their lives, work and volunteering, which could be exhausting.

The expectation that in any given disaster whether it be an MVA, or a house fire, a bushfire, a flood, there is an expectation as a first responder that you have to respond. There is the associated guilt if you don't. Particularly difficult for our rural communities who during times of drought can't always attend large‐scale fires because they're too busy running water or feed to stock. They're already sleep‐deprived. They've got no money coming in—particularly in drought because money is going out and paying for feed. The expectation is that they go out to fight fires and then there is the guilt if they don't. (Maise)

### 3.2 Unique Barriers With Rural Mental Health Services Hinder Mental Health Support

This theme explores the key barriers that impacted first responders' engagement with mental health services in RRR areas of Australia. These key issues and challenges included challenges with the nature of rural services, service accessibility, service acceptability, stigma and the perceived impact of identifying mental health illness. Issues with rural services were often reported by first responder and health care professional participants. With rural communities there is often a conflict between clinicians providing support services to first responders and having other relationships with these first responders. The likelihood of a clinician knowing the first responder outside their role was more pronounced in smaller communities and these dual relationships were seen as an overlapping conflict of interest that was difficult for both the first responder and clinician to navigate. This was described as a perceived lack of confidentiality by first responders who felt they could not access services in their local town and feared that seeking help would become common knowledge in the community. First responders reported attempting to access services from nearby towns to try and retain a sense of privacy. Participants reported that the boundary between professional and personal relationships was often blurred with first responder and health care professionals both highlighting a lack of anonymity in rural communities, which impacted their ability to keep professional lives separate from their personal life.

that idea that if you are seeking support on the ground, it's really difficult to know that you're going to not have any sort of overlapping conflicts of interest because if…If there's issues with seeing somebody in person, even if we know that psychologists and therapists are usually very ethical when it comes to not breaking confidentiality, I think that perception of knowing that there could be people in your community, seeing the same person can be a real barrier to people supporting that. (Marrisa)

In addition, rural communities often rely on fly‐in‐fly‐out transient workforce and health care services. This transient workforce has a direct impact on the community's willingness to trust and engage with both the first responders and health care professionals as they saw the services only as short‐term or a check box activity so the government could say they had services in place at the time. Health care professionals also highlighted that short‐term services resulted in lack of continuity of care and a lack of follow‐up as they were not allowed to engage with the community once their service had been removed from the area.

…certainly from talking with colleagues as well, that there is a level of distrust out there in those smaller communities that, oh you're just another service. How long are you going to be here for? So there's that kind of attitude as well. Having been involved with some quite large inter‐agency meetings, I am gobsmacked at how many services there are out there but there is a level of distrust because they come and go. Mine could be one of those [laughs]. The [non‐government organisation] I work for, which is tragic. I'm hoping not but—because there's so much work to do. (Dede)

There was a disparity in the support mechanisms available for rural first responders compared to their urban counterparts, which was recognised by both the first responders and health care professionals. For example, there was a perceived lack of resources available to support first responders' mental health and this had a negative impact on the first responders' sense of self‐worth and professional value. Another example was a lack of first responder staff which limited the peer support within their rural service, lack of experienced personnel available to respond to traumatic or critical incidents and reduced rural staffs' ability to take leave as needed (such as for mental health), often resulting in burnout and/or moral injury.

When you work in large centres, you can take a mental health day because there's a casual pool to replace you. In rural remote areas, if you don't work, you know full well that someone has either been called in on a day off or has had to pull a double shift to cover you. So how does – how does that actually help you take a mental health day to recover when you already know that you're causing somebody else issues? It's not just a guiltfree day to look after yourself. It's a considered, shit, if I take the day off, then that means Joey's going to have to work an extra day. The last time we spoke, she had this happening today and so if I take the day off, then she can't do – no, I better go to work because I don't want that guilt of causing her to do that. So that lack of a pool, that lack of human resources, or lack of available staff, means that you don't take that day off. (Laura)

There were several accessibility barriers that hindered first responders' engagement with services. For example, there were issues with the consistency and variability of the types of services available in each community, long wait times to access support services and the quality of the services with limited qualified staff to ensure services were delivered as intended. These barriers were particularly impactful for rural first responders experiencing a mental health crisis that needed immediate help. The lack of services in the local community resulted in first responders needing to travel to surrounding communities to access support. However, rural communities in Australia might be hundreds of kilometres away from one another. Travelling long distances to access services was identified as a barrier and first responders reported either not accessing help or waiting until they were in crisis before attempting to seek support.

Gaining access to support services that are proactive. The distances travelled to seek supports. The limited options available. Limited skill sets available within the community of providers. Difficulty accessing GPs due to waitlists. (Tabitha)

Other rural barriers identified by participants included the uncertainty that services would be available, siloed services, financial (cost of services, cost of travel to services) and time constraints (long wait times to access services).

It's just the large distances and getting support there and they're siloed….[organisations] they provide their own support. So it is very segregated in terms of if you're looking to access that support. I'm glad that it's there, but some collaboration between services and not holding tight or who's paying for what…(Jay)

Additionally, first responders experienced challenges with service acceptability. First responders raised concerns around a lack of trust for mental health support services in rural areas and a perceived lack of confidentiality and privacy when accessing rural service, especially those provided within their organisation, preferring to access services outside their workplace and community. First responders also highlighted that clinicians providing mental health services needed to understand both the first responder and the rural contexts, with some participants reporting that clinicians struggled with the vicarious trauma associated with managing first responders' mental health. The lack of understanding or experience in managing the impact of trauma on first responders' mental health added complexities to the issues in rural areas where there are limited services and large distances between services providers.

So not only do we blunt ourselves to our families and blur the lines between truth and keeping a nice happy story because you don't want to traumatise people you know… but then to have a therapist or counsellor or psychologist say, that's too much for me. You're like, oh, there's no one. No one can help me. That's happened to a fair few people. They hit a service. The service doesn't do what they expect it to do. You told me that if I went and got help that it would work …. No one told me that you'd have to shop around (Jay)

When disclosing mental health issues, both first responders and health care professionals reported perceived negative impacts on first responders' career, with limited access to return‐to‐work programs and organisations choosing to medically retire first responders with PTSD rather than invest in their recovery. Stigma was an issue raised by many participants, with the fear of stigma and the actual stigma associated with mental health in rural areas reportedly more pronounced and detrimental to the first responder than in urban areas.

How about we get rid of them and then we'll bring new people in. The new system, the total and permanent disability scheme is non‐existent now… The system now is such a way that there is no protection for the new ones [recruits]…it's just a recipe for disaster. They're that short on [first responders] that throwing people away prematurely, they've got a new rejoining team. You can rejoin, except if you've been exited with PTSD and stuff like that…. So, if you've got mental health issues then they will just, there's the door, off you go. (Simon)

### 3.3 Effective Mental Health Support Requires Greater Cultural Sensitivity to Rural Contexts

This theme describes the recommendations, initiatives, strategies, and resilience skills that have the potential to help rural first responders to better manage their mental health. Key areas were identified that require greater attention to improve mental health. One of these areas was empowering communities and people to manage their mental health better and strengthening the protective factors that already exist in rural communities. There was a recognition of preexisting support networks built into rural communities that could be used to support first responders' mental health. Strengthening these factors can help to empower communities, build resilience, and meet the gap between people's needs and service delivery.

I just think we need to identify what those protective factors are or what those strengths are and then invest into those, you know? So what is it that makes—what are families already doing that makes them more connected? What are individuals already doing that makes them more resilient? What are folks out in remote areas already doing? I think we can sit in ivory towers and pretend we've got all the answers and look at this from a deficit perspective, or we can assume that people have what they need inside them to be resilient and strong and empowered, but we need to facilitate that and get out of their way…Because so much of this is an empowering thing…. (Emma)

In addition, participants reported a need to provide access to mental health support for retired first responders (or first responders transitioning out of the organisation) and ensuring that mental health interventions are suitable and effective to first responders working in RRR areas. Participants described this as interventions being more effective and available; and consideration of the complexities that are unique to rural communities. There were some potential programs that both rural first responders and health care professionals identified that had potential to improve mental health, such as peer support, clinical supervision, debriefing, mindfulness and resilience programs. However, not one participant could report a positive, effective program they had themselves accessed. Participants did highlight there were some negative aspects to these interventions or strategies but mentioned a more preventative approach could help to alleviate this. For example, debriefing post traumatic incident can be re‐traumatising; however, a more preventative approach of undertaking debriefing routinely may be more effective in managing mental health.

Normalisation of mandatory weekly de‐briefings no matter what has or has not occurred during the preceding week. Usually, any debriefings that are offered are only done so retrospectively of an incident – meaning the bucket is already depleted. By normalising weekly sessions (even if they are only a few minutes of ‘nah, all good this week’) conversations for all staff, means that the staff have the opportunity to get familiar with a new (until embedded) process, thereby knowing that it will come around again the following week. Also means that familiarisation with mental health services occurs naturally in non‐stress environments prior to issues reaching critical mass. (Tabitha).

## 4 Discussion

The findings highlight several key issues that may shape rural first responders' mental health. Participants described trauma as having a significant impact on rural first responders that is compounded by rurality, including geographical isolation, smaller teams, limited resources, and higher workloads. They also highlighted how negative workplace cultures, a lack of recognition and unrealistic ‘hero’ expectations further exacerbate mental health strain. In addition, participants identified limitations in the availability of mental health support in rural areas, including inconsistent quality, limited first responder–specific trauma expertise, siloed service delivery and interventions not tailored to regional, rural and remote (RRR) contexts.

Many rural first responders experience mental health consequences due to the conduct of their role. As has been noted in disaster contexts, the psychological consequences of catastrophic events often outlast the immediate physical threat, leaving less visible but enduring impacts on survivors and responders alike (Li et al. 2026). In this current study participants reported experiencing vicarious trauma, anxiety, depression, isolation, post‐traumatic stress disorder (PTSD), suicidal ideation and burnout. These experiences are similar to the findings of previous research (Jones, Jackson, Ranse, et al. 2024). Other mental health outcomes identified in the literature include substance use, social withdrawal and emotional shutdown (Harvey et al. 2016).

These mental health experiences impact more than the first responder, unfortunately, as the responder brings these experiences home to their family, extending beyond the workplace and into family life. As a result, they can cause strain on family relationships, associated with domestic violence and have also been identified as a cause of intergenerational trauma for children (Lawn et al. 2022). In this study, participants described links between mental health difficulties, domestic/family violence incidents and increased divorce rates. These findings are similar to the findings of previous research. A qualitative review of family members' experiences highlighted that spouses may experience vicarious trauma, secondary trauma and/or overburden due to attempting to protect the family, with children experiencing higher levels of separation anxiety (May et al. 2023). It is often only those closest to a first responder who see the impact of the role on their mental health. Family members of first responders are often their greatest supporters, but their role often goes unacknowledged (Traynor et al. 2024).

Our findings suggest that in the rural context first responders can experience a sense of being celebrated rhetorically but unsupported materially. The expectation that rural first responders remain endlessly available and emotionally contained reflects what has been described as the ‘hero discourse’ in emergency services (Furness et al. 2021; Rees et al. 2022). While framed as celebratory, previous research suggests this discourse can also function as a disciplinary mechanism that simultaneously romanticises sacrifice and recasts work‐related distress as an individual weakness rather than a predictable response to structural strain. Similar to analyses of nursing work, emotional containment becomes a performance of professional legitimacy (McAllister et al. 2019) and our findings suggest this may be more particularly acute in rural environments where anonymity is limited and professional identity is inseparable from community surveillance. This also aligns with literature on moral injury, which suggests that trauma is compounded when workers are asked to inhabit heroic ideals without corresponding institutional protection (Thibodeau et al. 2024). Participants' accounts further reflect Rushton's (2024) concept of moral residue, described as the lingering psychological burden that arises when workers perceive their distress to be both produced and overlooked by the institution. Thus, workplace culture cannot be framed as interpersonal dysfunction alone but may be understood as shaped by systems that demand stoicism while normalising neglect.

Rural workforce research suggests that retention intentions are linked not only to rural background, but also to perceived professional opportunities and workplace experiences that influence morale and satisfaction (Seaman et al. 2026). Participants in this current study reported experiencing guilt around leave‐taking and this aligns with research on presenteeism in the rural health workforce (Ji et al. 2023), where scarcity of resources turns self‐care into an ethical dilemma. Rather than a matter of individual burnout, presenteeism in this context reflects what can be described as a moral economy of scarcity, where workers internalise responsibility for systemic under‐resourcing (Bundy 2024). In this framing, even taking leave for mental health support is recoded as abandonment of colleagues and community, making acts of self‐care appear morally suspect rather than professionally necessary. Within neoliberal health systems, this scarcity is reframed as a test of personal resilience, positioning self‐sacrifice as a professional virtue and obscuring the structural issues that produce the conditions in which harm to workers is caused.

Literature on rural health services consistently critiques fly‐in/fly‐out (FIFO) models for their impact on continuity of care and relational trust (Burnett et al. 2020). Our findings suggest that these workforce structures also carry psychological consequences as transient staffing diminishes therapeutic trust and contributes to what participants described as symbolic rather than substantive support. In this way, supports may be viewed as tokenistic, meaning that it is visibly present in policy but effectively absent in lived practice. For rural first responders, this sense of support being acknowledged rhetorically but not adequately available in practice likely intensifies feelings of abandonment, reinforcing a long‐standing pattern in which rural communities receive intermittent crisis responses rather than sustained investment. In addition, participants in this study reported confidentiality issues with local health care providers; travelling to seek help outside their community was a barrier to help‐seeking and FIFO options are also not ideal, which limited options to address the unique issues around service delivery and access. Hence, the model of service delivery needs to be rethought and a new approach developed that considers the uniqueness of rural communities, barriers to service access and delivery and is tailored to first responders' needs.

Our findings revealed several barriers to accessing mental health services that appeared to be unique to rural first responder contexts. In comparison to metropolitan areas, the availability of services was poorer and less consistent in RRR areas and participants noted that the quality of the few available services was limited by a common lack of expertise in the specific issues faced by first responders. These issues considerably limited the choice of accessible services, which was further restricted by confidentiality concerns and high levels of stigma (including fear of negative career consequences). Confidentiality barriers are known to be particularly impactful among first responders (Auth et al. 2022; Haugen et al. 2017), although these were further exacerbated by the nature of RRR communities, where there is a greater chance of being known to serving clinicians and greater fear of help‐seeking becoming known within the community. Similarly, while stigma around mental health and help‐seeking is generally found to be pronounced among first responders (Auth et al. 2022; Haugen et al. 2017), participants from this study reported that stigma was felt to be even more pronounced in RRR communities. These barriers collectively drove participants to prefer accessing support outside of their local community. However, accessing appropriate mental health care was hindered by participants' need to travel long distances to access support, a barrier that does not tend to emerge strongly for first responders in the wider literature (Auth et al. 2022; Haugen et al. 2017; Rikkers and Lawrence 2021). While internet‐based delivery can work toward bridging gaps in service accessibility and acceptability, for some RRR first responders, poor internet service limits the value of these service options. Therefore, while rates of help‐seeking are generally low among first responders (Kim et al. 2018; Rikkers and Lawrence 2021), the additional barriers faced by RRR first responders may increase the likelihood that help‐seeking is avoided completely or delayed until crisis point.

Participants' recommendations for improving mental health support for RRR first responders often centred on increasing and improving preventative or early intervention strategies, which supports recent calls to increase evidence‐based indicated prevention initiatives among first responders generally (Commonwealth of Australia 2018; Putica and Yurtbasi 2025). For instance, rather than conducting employee debriefing in relation to critical incidents, which has shown unreliable and potentially harmful effects (Ancarani et al. 2025), participants suggested that there may be benefit in organisations routinely engaging in meaningful mental health‐focused check‐ins that work to normalise discussions on mental health, assist in early identification and facilitate early help‐seeking among those experiencing mental health concerns. Peer support, such as Employee Assistance Programs (EAP) and resilience‐building programs were also prominent among participant recommendations for potentially helpful strategies, although emphasis was placed on the need for such programs to be well‐tailored to the needs of first responders in RRR areas. Best practice guidelines exist for providing evidence‐based psychological treatment to first responders (Bryant et al. 2024), although similar guidelines are lacking for prevention‐level interventions. An important consideration in provision of such programs is the emerging evidence that first responders are distrustful or less willing to use mental health supports that are provided through or funded by their employing organisation, partly due to confidentiality concerns (Marshall et al. 2021; Rikkers and Lawrence 2021). Perceptions of a mental health program or service as trustworthy, confidential and relevant to those in RRR areas appear to be key considerations for maximising acceptability and utilisation.

### 4.1 Relevance to Clinical Practice

Mental health is an important workforce issue that is relevant to many workplaces. If mental health support is not done well, we risk losing key workforce personnel because of mental illness, burnout, and decreased job satisfaction. This is exacerbated in rural first responder workplaces which are already under additional strain with reduced resources (including staffing), climate change, and additional economic pressures, so getting mental health support right is essential to ensuring we have a first responder workforce that is able to respond to the needs of our rural communities.

There are unique challenges that exist in RRR contexts that complicate provisions of mental health support and impact first responders' mental health. These contexts need to be considered by first responder organisations when planning and implementing services and support for staff and when developing and delivering mental health support services in rural areas. Currently, there is limited availability of interventions tailored to the needs of first responders in RRR areas (Jones, Jackson, Ranse, et al. 2024; Jones et al. 2025), as well as tested interventions for use with first responders in general that are integrated into usual routines rather than reactive after an event occurs to ensure a proactive, preventative stance is assured (Putica and Yurtbasi 2025). There is an urgent need to develop these specific interventions to include the development of digital interventions that are accessible in rural environments, including those that can be downloaded when internet access is available but do not rely on internet connection to access modules, which can be used more readily by people in RRR locations (Jones et al. 2025). In that way, RRR first responders will be better prepared and able to access services more swiftly when needed.

In RRR locations, services for people with mental health issues remain poor and there is an urgent need to address this paucity of service for all rural and remote residents. There is also limited qualitative research into the lived experience of RRR first responders; further research of this nature will facilitate a deeper understanding of the experiences of these RRR first responders and their family members that can be used to inform and design appropriate support services (Traynor et al. 2024). These findings reinforce earlier arguments that rural and remote first responders face distinct safety concerns and intensified mental health impacts due to limited staffing, resource scarcity and reduced access to specialised support (Jones, Jackson, and Usher 2024). As previously noted, reduced workforce capacity and increased workload in rural settings may heighten both safety risks and psychological strain, underscoring the need for context‐specific mental health strategies. Transition opportunities for first responders are also currently missing. There needs to be an urgent development of appropriate transition services to ensure RRR first responders are not subjected to further trauma as they move to retirement (Smith et al. 2022).

### 4.2 Limitations and Strengths

Our study attempted to recruit a representative sample of RRR first responders; however, our sample included predominantly female participants, despite emergency services often being male dominated, which may impact generalisability of the results. In addition, participants had worked in only two states across Australia (NSW, QLD), which may not capture the different challenges across different regions in other states. Nevertheless, the participants came from a range of first responder services capturing the essence of rurality and the impact of trauma on these FR roles.

## 5 Conclusion

This study highlights the complex and cumulative pressures shaping the mental health of rural and remote first responders. Trauma exposure alone does not adequately explain participants' distress. Rather, trauma operates within a broader rural ecology characterised by geographic isolation, limited workforce capacity, constrained resources and heightened community visibility. In this context, professional and personal identities often converge, intensifying emotional labour and reducing opportunities for anonymity, recovery and psychological detachment.

Our findings also illuminate how workplace culture can compound this strain. The persistence of ‘hero’ narratives, expectations of emotional containment, experiences of misogyny and discrimination and organisational neglect create conditions in which distress is simultaneously produced and silenced. Participants described environments in which being rhetorically celebrated did not translate into meaningful institutional protection or support. Such dynamics shift responsibility for coping onto individuals while obscuring the structural conditions that give rise to psychological harm.

Importantly, service limitations further exacerbate these pressures. Inconsistent quality of care, limited trauma‐informed expertise specific to first responders, siloed service delivery and a lack of interventions tailored to rural, regional, and remote contexts reduce the likelihood of timely and effective support. Addressing rural first responder mental health therefore requires more than resilience‐building initiatives or individual coping strategies. Structural reform, culturally responsive workplace practices and coordinated, context‐specific mental health services are essential. Recognising the layered and systemic nature of these challenges is a critical step toward meaningful change. Without confronting the organisational and structural factors embedded within rural emergency service environments, efforts to support first responders risk remaining symbolic rather than transformative.

## Author Contributions

Study design: Rikki Jones, Kim Usher, Debra Jackson, Kylie Rice, Jamie Ranse, Clare Sutton, Aimee Gayed, Andrew Arena and Lisa Clegg. Ethical Approval: Rikki Jones, Kim Usher, Debra Jackson, Kylie Rice, Jamie Ranse, Clare Sutton, Aimee Gayed, Andrew Arena and Lisa Clegg. Data collection: Rikki Jones, Kim Usher, Debra Jackson, Kylie Rice, Jamie Ranse, Clare Sutton, Aimee Gayed, Andrew Arena and Lisa Clegg. Manuscript preparation: Rikki Jones, Kim Usher, Debra Jackson, Kylie Rice, Jamie Ranse, Clare Sutton, Aimee Gayed, Andrew Arena and Lisa Clegg.

## Funding

This research is funded by a Medical Research Futures Fund (MRFF) grant MRF2031244.

## Ethics Statement

University of New England Human Research Ethics Committee HE24‐066.

## Conflicts of Interest

Co‐Author Prof Kim Usher is the Editor‐in‐Chief of the International Journal of Mental Health Nursing. COREQ: The authors confirm the COREQ guidelines were followed for reporting of this research project.

## Supporting information


**Table S1:** Selected addition quotes.

## Data Availability

Data is available upon reasonable request though the corresponding author.
