# Supplementary material for: Mental Health of First Responders in Rural Australia: A Qualitative Exploration of Intersecting Individual, Organisational and Contextual Factors on Psychological Wellbeing
Source: Int J Ment Health Nurs. 2026 Sep 8;35(5):e70349. doi: 10.1111/inm.70349 (PMC13554821; doi:10.1111/inm.70349)
Supplement: Supplementary file 1 [file INM-35-0-s001.docx]

Selected addition quotes: Table S1

| ***Theme One: Rurality further complicates trauma and mental health*** | | | | |
| --- | --- | --- | --- | --- |
| ***Subtheme 1: Rurality impacts service delivery & ethical challenges*** | |  | | |
| Ethical Challenges | Karie- Especially when you live in such a small community and you are accessing services, you can’t necessarily just go to the GP, because everyone else will know. Everyone in town will know within the next 30 minutes that you were at the GP or you went to see the psychologist, regardless of how sneaky you try to be. Obviously, there are lots of telehealth services, but, yeah, I don’t know. That’s my opinion. But I like to hope that people would. | | | |
|  | Suzette- We have mums and dads. We have about four families where the cadets are there and then they’ve got the young adults in the juniors, then they’re in the adults. So when they go on duties it’s a whole family affair. They’re all at the first aid post all experiencing the same trauma on different levels. So how are the kids coping? Who is debriefing this family? Are we doing family debriefs and all the stuff that comes with it? Then if they live in a rural, remote area, and the likelihood is if they are living in rural or remote, they will come as a family because they’re not going to leave the kids behind. They will do the activity together and if it’s something very traumatising, like a motor vehicle accident, or - being on duty, we’ve had gorings and stuff like that in some of the rural shows, and the children are looking at it. Watching it. Watching the method of injury. So, yeah, all of those kinds of things will compound as well. | | | |
|  | Maise- I think the expectation for people to always be there to fix things and it doesn't just have to be trauma. It has - Australia has a great volunteer history. It’s one of our greatest assets, is our need to help. I think all disasters clearly indicate our skill sets, and our strengths as Australians to roll up our sleeves and pitch in. There’s also the expectation from state and federal, and government services, that because we are free, we’ll do everything. We’ll go outside of our realms because we are a free service. | | | |
|  | Maise-I think the other people are very wary - our partners and our family are very wary if they’re not in the service. Imagination can be a wonderful and a freaky beast all at the same time. The not knowing is worse than the knowing sometimes, let alone what they have to deal with when we come home. We can’t always be easy people to live with, I’m quietly confident of that. I think the only one that wins out of some of this stuff is probably Dan Murphey’s. I think they’ve probably got the biggest benefit out of everything. Yeah, they’re things that weigh on the family and people constantly seek them for updates to information so they feel like they need to know more than what they probably should because the expectation is on them. When people say, oh, look they’ve gone out to such and such, what’s going on? It’s challenging. | | | |
|  | Marissa- I would agree with that, and I’d add, I think in rural and regional remote areas, people’s exposure to climate change and associated disasters is just – unfortunately it feels like recently it’s through the roof. But in particular I think it has such implications for their quality of life or the systems in place that are supposed to support them, and just day to day life. If you’re thinking about areas that have been affected by fire. Areas where roads have been damaged and people can’t get to schools for months and months. It impacts their childcare. It impacts their neighbours. Even sometimes people have lost their own homes. | | | |
|  | Karie- To be honest, I don’t know. I’ve had many years with New South Wales Health, so I don’t know what adequate support would look like because I think it’s very dependent on the job, the community that you’re working in, because each community is so different in what they have available. The stories behind each community are so different, so what support looks like I think needs to be variable depending on where you’re going. If you go and work in B[name removed], what you’re exposed to would be vastly different to if you’re working in other areas. But it doesn’t mean that they don’t require support in, I don’t know, [unclear] versus [name removed] but what they need, I feel, would be different because they’re exposed to different things. Their level of [unclear] support there, it’s just not there. | | | |
|  | Sharn- It does impact on us. You feel guilty particularly around the Christmas time to go away. It’s the most hottest period to go, to go away for a holiday or time out, and you really know that there's hardly anyone there on call, and it’s just supporting it. You feel obliged and yet you shouldn’t feel obliged because you're only a volunteer and we actually do have the paid people there and I suppose maybe this is how they're keeping their costs down too because here’s the volunteer, you don't have to pay them wages or anything, but I'm telling you now, it’s flipping exhausting. It’s exhausting being on that hill and you have to get a fricking chainsaw and you’ve got to clear that track and there's only two of you who are trained to use the chainsaw and they're so heavy, like someone my size to lift a chainsaw in the first place, and you’ve got all your gear, you’ve got a helmet on, you’ve got your jacket and your boots, it is stinking hot, you're hoping like hell the helicopter comes and just dumps a big load on it just to get rid of it, but you know that’s not going to happen. It’s just really exhausting and it’s really hot. | | | |
| Service challenges | Maise- So sometimes it’s a really powerful thing, and other times there’s the flipside as well. So you have isolation in terms of poor education, and that limits their ability sometimes, and it limits their - it increases their sensitivity to working with other people if other people find out that they can’t read or they can’t write. So - but they can hang on to the end of a fire hose, they can drive a fire truck, they can be an amazing first responder. Literacy doesn’t have anything to do with it. So there’s that sort of dual flipside as well for some of our rural and remote communities. | | | |
|  | Maise- So there’s pressure on the staff, there’s pressure on the communities, there’s pressure on the rural and remote responders, and it’s something that - we’re pretty good at finding solutions but we don’t actually have a solution for this. It’s becoming increasingly difficult and increasingly stressful. So that’s a big stress at the moment and nobody really has an answer to that. At the end of the day, the government can’t afford to pay our volunteer services. That’s a fact. That’s why they get used so much because we are free. At the same time, we do have to be very, very careful as a society that we don’t burn that out and we don’t abuse that. Yeah. | | | |
|  | Emma- They need to be explained in person. They need a face to those. They need people to spend time with them. We don’t get that time, right? Or if we do, we have to make the really difficult decision of which one of these report areas are we going to prioritise. Again, the moral injury around that for the professionals is huge and then the lack of sufficient services for people on the ground is equally as huge. Because there’s just not - that’s just not how we’re funded. I think emergency services, there is so much emphasis on technical capability, as in the technical response, not the people response. | | | |
|  | Laura- So I’m seeing a – I’m seeing a graph in my head but trying to word the graph. The more rural, the more remote, the more acute the presentations. So that’s not an inverse relationship – that’s a direct relationship. However, the more rural or remote, the more acute the presentation, the less stable the workforce, the more you rely on fly-in/fly-out. So therefore you don’t have the familiarity with the patient – so there’s an inverse relationship. By not having that familiarity with the patient, the more acute. Because something that may seem minor to a fly-in/fly-out worker, actually presents red flags to a clinician who is familiar with that patient. So there is, I believe, a direct relationship between remoteness and acuity – now I’m still talking patient acuity at this stage – and resilience [slash] – I won’t use the word resilience, yeah – and familiarity with the fly-in/fly-out workers. Now based on that inverse relationship, the mental health issues for those first responders gets worse the more remote. | | | |
| ***Subtheme 2: Workplace factors exacerbate mental health issues*** | | | |  |
| Support Mechanisms | Karie- For me personally, I’ve both worked in emergency departments across rural remote locations, and in some areas we are the first responders. Obviously, I’m not doing that role anymore because I’m now working as a mental health clinician, but in my experience it’s both the vicarious trauma of stuff that we’re exposed to that, when we don’t have adequate support mechanisms in place, can be quite detrimental to mental health. The isolation of working independently and not having, again, that support network would be my two. | | | |
|  | Laura- You can’t station a mental health nurse, psychologist, or psychiatrist in every single organisation permanently. There is no easy answer to the relationship between lack of familiarity, or staff turnover, and improving their resilience from a mental health perspective. Given the story I just told you to question 6, it took for me to move to this role, move to this community in August last year, for me to stop seeing flashbacks about that one particular instance I just described… two years prior. It took for me to step out of a first responder role and into a nine to five role, for me to be able to start to recover from that one particular instance. Therein lies the lack of staff. Because both my wife and I are incredibly skilled remote area nurses, and incredibly resourced in internally resourced remote area nurses. Heavens, she was a psychologist before she was an RN. She’s incredibly resourced internally. Yet we still had to go, enough is enough – we cannot be doing this. | | | |
|  | Tate- Once again, comes down to cultural stuff. We can't spare you, we can't get someone in to cover you, you just got to basically suck it up, keep going. That's the big one. Plus, they've been able to go, yep, we're going to put you off the road. Take a day off, take two days off, or if you want to make an appointment, we're going to get you to talk to a peer or a psych or whatever. | | | |
|  | Tabitha- Yes, social isolation. Available resources. Available funding. The cost of accessing services. The training of practitioners that are available to provide supports, that aren’t trained to support people that have experienced significant traumas, specifically within a work setting where traumas are repeated, ongoing and accumulative. | | | |
| Organisational issues/culture | Lucas- it needs to change. Unfortunately, even our suicide has gone up, especially in the policing segment as well. There’s just the head in the sand stuff that frustrates me, that’s why I’m glad I walk away now from trying to solve these problems. But yeah, nothing is going to change. Recruitment is going to remain - I don’t know what nursing is like but police can’t fill classes because the word’s out there that you’re not going to be looked after, so why would you send your son or daughter and encourage them to go do it? I certainly don’t. It needs huge, huge organisational improvements. | | | |
|  | Karie- I agree. Paramedics rates are going down, nursing rates are going down, as you said, police rates are going down. We’re already under these massive workforce shortages across Australia, and the world, I suppose you could say. I don’t think it’s going to ease up any time soon, which is adding to that mental load and burden because you end up having to do all these overtime shifts and pulling doubles and working more or they’re cutting corners to make sure that they meet their minimum requirements as an organisation. Which then, obviously affects the on-the-ground person, which is your first responders. Something that needs to change, as Lucas said | | | |
|  | Emma- Then I guess, I would also just being within an organisation, sort of add the organisational factors that contribute to mental health. I think often we think about operational factors, you know, the trauma, the exposure, et cetera, but the moral injury within first responders is huge. So if you’re in a town and this is your world but you’re seeing your morals - if you have to go against your morals or you see people acting in ways that you don’t agree with, but you don’t want to leave because it’s your world. So then you have to stay within a context in which you’re being morally injured and that can go for decades. | | | |
|  | Emma- So that moral injury manifests a little bit differently than say PTSD or post-trauma kind of symptoms, but it’s a really significant part. So I think that sometimes if there are issues within the organisation - those organisational pressures. Whether that’s lack of resources, moral injury, feeling like the agency doesn’t have your back for whatever reason especially if you’re in a rural area, all of those things I think accumulate to create quite significant mental health issues for people for whom first responding is their life. It’s their community. It’s their identity. It’s their everything. | | | |
|  | Emma: [Laughs] It’s so true. We get such warm welcomes whenever we - for the most part when we visit rural and remote areas and members. The thing is, we are just not staffed to do that kind of work. That’s the reality of it, you know? Then the other flip side is that you then get this team of well-being professionals who get incredibly burnt out and all this moral injury because they’re trying to do things in a best-practice way. Which is to go and spend time with people face-to-face, where they are, to help build a bridge to all of these support services that do exist. | | | |
|  | Tabitha- They have to be boundaries in doing the role when they’re on site with people. They have to stay professional within that role. Outside of that, when they’re in the car or when they’re with colleagues, they can talk about what they need to talk about, if that’s where they feel comfortable. But outside of that or the station, it’s sort of their personal life that that’s coming out, if at all. | | | |
|  | Emma- I know it doesn’t seem as interesting to talk about and it doesn’t have fancy names but that’s where we’re going to make the impact. So much of this stuff could be - I’m not going to say fully prevented. I don’t think you can prevent mental health. I don’t think that’s true. I don’t like that word even. I think when people say they’ve got a prevention program, I don’t think they do. I think they have a preparation program. I think they prepare people to meet the demands of what they’re facing, but you cannot prevent mental health. You can prevent harm, and that’s different. So you can - when you prevent harm to the best of your ability or minimise harm to the best of your ability, that’s actually - and if we look at it, that’s the responsibility of all leadership under the WHS legislation. That’s what they’re meant to be doing, right? If we look at that hierarchy of control, so what works the best when it comes to mental health, training and support services, EAP and psychologists are down the bottom. They’re down the bottom of effectiveness. Up the top is all of our systems-level stuff as well as things like leadership training, and empowering people on the ground | | | |
|  | Emma- say that it makes me feel like there should be more of a focus on remote and rural areas as first responders in leadership positions. Actually what today has taught me is that we need a seat at the table. Specifically, I don’t need a seat at the table, I think the members that I serve need a seat at the table. So if we saw more rural and remote first responders who have actually had lived experience first responding being in leadership positions, both within the agency and outside the agency, I think that would make a huge difference to have their voices be heard | | | |
|  | Lucas- If we’re talking about the care for police within management, that was pretty horrible and that’s seen throughout the state now and there’s consequences, obviously, as a result. But that’s also too because they are left on their own devices. You can say what you want as far as a business charter goes in [City removed], but then once regions are running their own places or their own stations, then they can just tick a box and tell someone in [City removed] that they’ve done those things. But the actual care and attention for those that are on the ground, it doesn’t happen and it’s still continuing even to this day. Even though I’m out, I still hear stories. | | | |
|  | Emma- I think it’s getting harder because now there is an increased demand for the professionalisation of volunteer services and in some ways that’s fantastic. We’ve got so many more programs and all of this infrastructure in the agency, but it puts more administrative burden on the frontline volunteer. They get so many more emails than they’ve ever gotten before. So many demands on their time over and above operational demands. | | | |
|  | Jay- So it's just different in terms of the restrictions that are there for career, the physical constraint against you and yes, you could quit. Everyone's like, well, if you don't like it, you can quit and you're like, yeah, I know, but I went to university, I studied, I committed to this, I wanted to be in the job. So then you end up staying out in regional and remote areas and you're like, I don't really want to be here but getting to the beaches is like people have to die. So yeah, I definitely think those factors play a big role in added mental health stresses that aren't necessarily associated with being in metro. Metro have forced overtime and don't get a lot of breaks but then they get the social side of things, which is massive. If you're not part of that social thing, social scene or group, then you can feel lonely in that area too. So it's not just a - yeah. Did I answer the question? I feel like | | | |
| Workforce & Professionalism | Maise- the other interesting thing is that all of this stuff, regardless of what service we roll in, is based around technology. So you’ve technological literacy but even more basic than that, there’s actually the ability to be connected to technology. How many times do you go down the road and you don’t have any phone? You know, and we’re going to put in these fantastic terminals. Great. On the dashboard of your truck. Fantastic, but if I can’t get my phone to work, then how am I going to get the computer, or a laptop, or a tablet to work? Then what do you do? Then we’ve got to go back to being rural and remote and tapping into those skills. So all of this stuff is being pushed down to us from training through to forms, through to administration, through to more and more things that are being - on vehicles, on response vehicles, that rely on our network connectivity. If we don’t have a reliable network connectivity then what do we do? This is where our older members come into the fore because their skill sets were around long before even pigeons had notes strapped to their legs. | | | |
|  | Laura- So I don’t know the answers, and I really hope this research project can start coming up with some. Because all of us out there need it somewhere, somehow. Or we will just keep losing staff, just like we’ve had to take a step back from it. There’s an SES in town here – I’m not joining it. The amount of time, and money, and effort, and education, and years that it takes to get the experience and the qualifications, and the knowledge, and the background, and the confidence, to do this role in the first place, for people consistently to go, wow, enough – I can’t do it. They – they, being the different organisations – are losing such a wealth of resource continually. Because primarily the mental health of the human resources, the first responders, cannot – you just can’t keep giving out of an empty bucket. So you walk away instead. | | | |
|  | Sharn- Yeah, I think we take on too much. We’re suddenly not working and we've got a bit of time so it’s like, hey, I'm going to join this group, I'm going to join that group. You know what? You're taking on too much. One’s enough. It’s exhausting because within these groups, they also expect you to do online training which, if you’ve ever been to Quirindi, our internet service is absolutely just shocking. There was a time when the only time our mobile phones would work was if we went and stood near the chook shed outside. It’s just shocking. So they expect you to do online training, they expect you to go away and do courses like on the weekends or whatever. Well, a lot of us still actually work, like we've still got jobs, and it’s juggling those times. These courses aren’t just a one-day thing; they're like this weekend and then the next part of it is the following weekend. Well, that’s a big ask to ask work off, oh, look, I need this weekend off and I need that weekend off out where we live. | | | |
|  | Jay- I don't know. I feel like maybe that siloed - because you don't know. If you're new you just go along with things. If you've got an older style - it depends on your style of manager as well. For example, I had someone break into my house when I was home and I had to lock myself in the room. There was no one home. I was single, on call. I had to wait for the police to come and chase them out and things like that and that was quite traumatising for me. My manager's response was you still need to do on call. You still need to keep doing what you're doing. I said, should I report this and they said, no, don't do that. Just I need you on call because you're the only one in a 300-kilometre radius that can respond. I'm like this young 20-year-old in tears, just emotionally distraught, away from everyone. I was up at the nursing quarters because I had nowhere I felt safe, so I just stayed in the ambulance. One of the ladies caught me and scared me and she's like come in, we'll give you the palliative care bed and opened up her doors that way for me so I could feel safe where I was. The managers just didn't see it as a big thing. It was just a, oh yeah, you get broken into, it's fine. This stuff happens. It's part of the job, you'll be right. So that attitude I guess, of not acknowledging the individualised responses of people to mental health concerns. | | | |
|  | Jay- I need to wake up at the drop of a hat. My sleeping is affected because when you do on-call and you're getting woken up, if you don't get a call, you check your phone to make sure that it's still on and you don't know what you're getting called to. So you're in an environment where you could get called to anything. Your clinical level's not fantastic in terms of your own abilities and perceived abilities. So your confidence is low, you're not sure where you are, and if you haven't grown up in the country, it's a brand-new effect for you and you feel trapped. There's no other word for it. | | | |
|  | Karie- So the isolation geographically but also socially, especially when you’re on call 24/7 for seven days. You can’t leave, you can’t go exploring or adventuring or whatever, you get so far removed from other networks there. Especially if you’re partnered with someone that you may not necessarily get along with because it’s literally the two of you and too bad, so sad you’re on for seven days and you can’t call in sick. Well, you can, but it's that perception that you can’t call in sick, you can’t have a day off for those seven days. Similar with the nursing side as well, I’m not 100 per cent on police but I think it was similar. There’s no one else. You’re it. | | | |
|  | Karie- I agree. Paramedics rates are going down, nursing rates are going down, as you said, police rates are going down. We’re already under these massive workforce shortages across Australia, and the world, I suppose you could say. I don’t think it’s going to ease up any time soon, which is adding to that mental load and burden because you end up having to do all these overtime shifts and pulling doubles and working more or they’re cutting corners to make sure that they meet their minimum requirements as an organisation. Which then, obviously affects the on-the-ground person, which is your first responders. Something that needs to change, as Lucas said | | | |
|  | Jay- So the investment and making your social network - I had quite a good social network there but it was very difficult because I couldn't go beyond 10 kilometres of the town, which meant that all the social events, the gatherings on the farms and things like that, I couldn't go to because I was restricted by my duties. Even when I wasn't on shift, I was on call. So the on-call means that for seven days, 24 hours a day, I'm property of Ambulance, I'm not property of myself. I need to wake up at the drop of a hat. My sleeping is affected because when you do on-call and you're getting woken up, if you don't get a call, you check your phone to make sure that it's still on and you don't know what you're getting called to. So you're in an environment where you could get called to anything. Your clinical level's not fantastic in terms of your own abilities and perceived abilities. So your confidence is low, you're not sure where you are, and if you haven't grown up in the country, it's a brand-new effect for you and you feel trapped. There's no other word for it. | | | |
| Rural Connections | Masie- Getting people to reach out and connect is always challenging. Particularly if you’re dealing with a lot of men whose communication skills and confidence may not necessarily be there. Women are a lot better at networking. Men are getting better, but we still have a cohort where they go home, and they go back to their farm and again they’re isolated. So who do they talk to? I think Susan is correct. Our peers, they are people who were there. They know what you saw. They know what you felt. They understand not sleeping. They understand not being normal is normal. | | | |
|  | Emma- Then I also think there are a lot of other diversity groups that we need to consider. There’s obviously age, but, you know, we think about disability for example, and different of ability and the need to be able to find meaningful roles and connections for people who have varying levels of ability and that could be physical, mental, or intellectual. So we really need to think about how being in first responders can be such an empowering and important part of their experience, but it can also trigger some of the underlying vulnerabilities that exist for those members as well. | | | |
|  | Suzette- I’ve experienced our cadets - the cadets that we’re attracting at the moment, quite a number of them are neurodivergent or have gender issues. They’re coming in for that sense of - one particularly with the ADHD cohort, where to use up some of their energies and gain a hyperfocus in many ways. They do tend to hyperfocus on their volunteering, and then how do you manage that and the leaders who are not equipped to handle neurodivergence in the group? Then, how does that neurodivergence, as was said before, compound the issues of - particularly with their emotional dysregulation and the first response situation? Because they feel the sadness quite a bit deeper, they feel that anxiety of not being able to do something in a particular situation stronger, and how does that then impact on their mental health and well-being? | | | |
|  | Ruth- Yeah. I think there is, but I don’t know the reason. But having been in an organisation that has 100 per cent paid employees basically - or 98 per cent – and then going to an organisation where it’s about 80 per cent volunteers or 90 per cent volunteers. I have no university research study behind this statement. But I think it’s because they’re doing other things. Their whole life is not dependent on their salary with that organisation. Getting a promotion. Feeling that they have conflict in the workplace and things that – volunteers have a lot of life outside their volunteering. Although some of our volunteers donate many many hours per week. But there’s not the same pressures on it. They do it because they love it, and they want to help the community. I’m not saying that that’s not for other first responders as well. But they have a job usually and they have some sort of form of employment. So, I think their life – and I don’t like this term – but it’s much better balanced by being a volunteer. I think they get a lot of satisfaction just out of being a volunteer but it’s not the be all and end all of providing for everyone in their family for example. | | | |
| **Subtheme 3: *Rurality complicates the impact of trauma*** | |  | | |
| Mental health issues in rural first responders | Tabitha- Yes, exposure to potentially traumatic events. Moral injury. Difficulty in the workplace. They’re different because there are not the same amount of staff responding to the different jobs that come up. So it’s the same staff members attending multiple jobs per day. So repeated exposure. | | | |
|  | Tabitha- I think it’s when people are posted to regional [name removed], they’re usually away from their family connections as well, so there’s a lot of isolation in that respect. So family members that live away don’t tend to have as deep an insight into that person’s behaviour going forward, because they’re not there to see it every day. So the changes in someone that’s come independently up here are only being monitored through the Department and by the people that are around them, and not their immediate family, which are probably the best people positioned to make those observations. That’s what I’ve noticed in the role here. Again that social isolation, because you’re away from your family and that network is another stressor for people as well and probably just shifts and the length of travel around, to just get jobs done as well. You’ve got that fatigue coming in. | | | |
|  | Simon- I broke down in my supervisor’s office in front of all my work mates, and that’s the last time I stepped foot inside [Town removed], and it was so embarrassing. Then I had to go through all the process of get a WorkCover [unclear], get this, get that. That’s not what you’re thinking if you’ve got - developing PTSD, you need access right now. | | | |
|  | Jay- Then within the organisation I think there's always been this fear of repercussion. So a stigma and it's associated with job progression and advancement because you've been tainted somewhat. I don't believe that to be the case at all having taken time off myself for PTSD and coming back was actually not rewarded, but the people calling it brave. That was three or four years ago for taking time off and then coming back. | | | |
|  | Lucas- It needs to change. Unfortunately, even our suicide has gone up, especially in the policing segment as well. There’s just the head in the sand stuff that frustrates me, that’s why I’m glad I walk away now from trying to solve these problems. But yeah, nothing is going to change. Recruitment is going to remain - I don’t know what nursing is like but police can’t fill classes because the word’s out there that you’re not going to be looked after, so why would you send your son or daughter and encourage them to go do it? I certainly don’t. It needs huge, huge organisational improvements. | | | |
|  | Emma- So when the organisation is not doing what it needs to do, for whatever reason you know, I think that that has a really significant impact on members. Again, I feel like the further out you are the more compounded those things become. Because it’s up to you or no one else. There isn’t - there aren’t other services. There aren’t other units. There aren’t other members available. So, yeah, you have that feeling of burden of responsibility so you can’t take your own self-care seriously. Then that’s where the burnout happens | | | |
| Impacts on first responders livers | Lucas- Statistically, all first responders are at huge risk of divorces, alcoholism, everything major that kills us in Australia, first responders are likely to be impacted by. It’s pretty sad, the statistics. But still, the same things are happening over and over. That includes marriage breakups, domestic violence, all those types of things. It’s a huge burden because you could have come home from something really, really horrible and you walk through the door and it’s a whirlwind in your family life. Your partner or your kids need to download what’s happened to their day because you’re that carer and supporter, but then suddenly you’re not present or you’re reacting quick to anger - that was my huge one. So yeah, it’s a massive, massive impact on family. | | | |
|  | Tabitha- Okay, it impacts families - there’s a lot of withdrawal from families. Self-isolation. They find it difficult to return home some days. They want to be alone. There’s domestic violence that happens. Reduced contact with children. High rates of divorce and failed relationships. Difficulty actually making time because of shift rotations, to be present with family. Yeah, I guess the family is relied upon to provide that backup for everything. Sometimes they don’t get their needs met as well, because that threshold for distress is a little bit higher in a first responder. | | | |
|  | Emma- …it’s - look and then - I think there are few things here. Sometimes there are multiple first responders within the same family. So I think that’s one thing. So for our members, a lot of them are living with family members with PTSD, for example, and so you’re either a carer of a family with that or you, yourself are experiencing some kind of post-traumatic symptoms of some kind. Then when we think about the manifestation of those mental health issues at home, it can look like drug and alcohol use, it can look like withdrawal from the family unit, it can look like domestic and family violence. | | | |
|  | Lucas- Personally a huge impact on the family, not that I was aware of it, but you don’t realise you bring a lot of it home. It might be the emotional outburst from seeing lots of traumatic stuff that affects the family, the wife and the kids as well. The lack of sleep, that’s a huge thing. You might be present but you’re not present. The frustration with the ongoing change of rosters has a huge impact on the family as well. Statistically, all first responders are at huge risk of divorces, alcoholism, everything major that kills us in Australia, first responders are likely to be impacted by. It’s pretty sad, the statistics. But still, the same things are happening over and over. That includes marriage breakups, domestic violence, all those types of things. It’s a huge burden because you could have come home from something really, really horrible and you walk through the door and it’s a whirlwind in your family life. Your partner or your kids need to download what’s happened to their day because you’re that carer and supporter, but then suddenly you’re not present or you’re reacting quick to anger - that was my huge one. So yeah, it’s a massive, massive impact on family. | | | |
| ***Theme 2: Supporting mental health requires overcoming barriers*** | | | | |
| ***Subtheme 1: Barriers to engagement with mental health services and support*** | | | |  |
| Service Acceptability | Suzette: I think there is a thing about EAPs. That rural people don’t know who they’re talking to on the other side of that phone and therefore they are less likely to access the EAP. They might go to Peer Support. They might go to their chaplain if they know the chaplain because the chaplain is on duty with them. I mean the issue with chaplaincy has always been, unless you’re prepared to be on the front line with them, they’re not going to connect to you because you’re a stranger. | | | |
|  | Maise- Some of our community still haven’t been spoken to by these professional services. So the level of distrust and damage that that causes is quite significant and is much harder to soothe and to get them back on to the track for healing. Why aren’t they accessing help? Because frequently in these communities it’s not available, or they’ve got to travel. Or now with Medicare and stuff, they can have six free visits but then after that, you’ve got to pay. How long is the waiting list to get in? What if you strike somebody who really you can’t connect with? It’s not like you have a huge array of choices to go and see another practitioner or another counsellor, you know? You don’t always connect with the person that you first are paired up with. You’ve got to work hard, and you’ve got to be confident enough to fight and say, look this isn’t working for me. Is there somebody else I can connect with? | | | |
|  | Lucas- Outside of the police to try and get mental health care – and I think this is just the same for all first responders – there’s a difference between getting someone who’s super keen, fresh out of uni that has studied psychology and then getting someone who actually understands. So a lot of the people that seem to go within the field will go into quite a busy area, that seemed to be the Sydney areas or at least the city areas, because they can get experience. But those that do come out here don’t seem to have the experience and there’s a big thing for trust for police to disclose anything in regards to their mental health because they don’t trust the person that they’re speaking to | | | |
|  | Laura- Because it’s too detailed to try and explain in a half-an-hour consult, or an hour’s phone call. So first responders, in general, not just because I’m making a generalisation because my experience [says it was it], but a lot of the first responders, so colleagues that I’ve spoken to, have had the same thing to say. I’m not just talking to some stranger and having to repeat everything – I just won’t do it. So therefore they don’t seek help. | | | |
| Service Accessibility | Marissa- They end up having that real sense of being let down because, just like with your GP, if you have to wait six weeks for an appointment, you’re probably not going to be that keen by the time it rolls around. A lot of the time people will cancel after that kind of protracted waiting time. Especially if they might have had quite a high threshold to reach out and they might be in a more [serious] state than they’ve kind of realised. | | | |
|  | Lucas- As far as access to mental health resources, there’s some fantastic programs that are, again, set up in [city removed] and I had the pleasure of working a few in [city removed] as well. But once you, again, get past that border, you don’t really have access to that. Even programs they tried to set up that was a rural and remote program, that was working with the [city removed] guys, that was shut down pretty quick. | | | |
|  | Jay- There's either an overabundance of information coming out or not enough or a bit of saturation. So you're a bit lost as to where to go with that. In rural communities it's probably a bit more isolating for the fact that they know that there's other stuff that they can access but not 100 per cent sure what that actually is online, for example. So then it means you have to come through your GP but if you don't live out there - so for example, I was placed six and a half hours away from my home and my doctor of over 20 years since I was born. | | | |
|  | Jay- If I had to make an appointment as a healthcare professional in a small town of very - it was a remote town that I was working in, you don't want to access those services because you go to the doctor for mental health issues or you go to the hospital and it's a very small community and that level of safety mixed with professionalism and access is very difficult. I was out there about 10 years ago now and I guess a lot has progressed since COVID and changes in that space that there are a lot of online services and phone calls and things that can be made just externally on your own as an individual. | | | |
|  | Tabitha- Gaining access to support services that are proactive. The distances travelled to seek supports. The limited options available. Limited skill sets available within the community of providers. Difficulty accessing GPs due to waitlists. Stigma, a tight stigma within the community, within the population of employees. I can’t think of anything else off hand. | | | |
|  | Ruth- Whether that distress is – looking at policing – whether it’s anger by the alleged offenders or whether it’s violence in the home, or car accidents, et cetera. Then the ability and wait times to get in to see some of those support services I think impacts them as well. | | | |
|  | Maise- I think that’s where we need to start plugging in our investment because we don’t have mental health services to stretch far and wide. Our mental health services, we know we do a band-aid job, and we go home at the end of the day worrying about all those people that we interacted with. Where are they at? How are they managing? I can’t help them heal. I can only give them a band-aid and I’ve got to hope that that’s enough. So how do we get these skills out into our community? Because in our rural and remote communities, it is our strength. Shotguns or no. We all need defence mechanisms but it’s our communities, it’s our people, which embrace our people and we have to move through these events together. | | | |
|  | Ruth- We spoke about financial stresses and things like that. Some of it, it does come back to the federated world in which we live in of State versus Commonwealth. Marissa probably has more understanding of this than me. But it was certainly an issue that came up in some of our discussions about the Medicare rebates and different things of what they were entitled to be able to keep them either mentally healthy or help them if they had mental health issues. | | | |
|  | Jay- There is the barrier of cost of services. So for example, I don't know if you can use this, but when I see my psychologist, I've got a mental health plan and it was when you had 20 and then I had 10. I make sure I regularly use those just to help me. Seeing a private psychologist using that, it was $280 an hour. I would get $100 back. Using my private health, I would get 180 back. So because I was paying for - so I use a combination of both. I use my private health first until I'm maxed out and then I use the mental health plan. So I found someone who's amazing. She's highly individualised where she is and I require that, but I have private health insurance. How much refund you get and how much services cost, just because they're there doesn't mean they're affordable | | | |
| Perceived impacts of identifying MH issues | Jay- It's part of your mental health. You're going to burn out if you don't. You're saying I'm just over it and there's a reason for that. If you take leave and are proactive in that space and have the opportunity, you can start to dwell on what that is. That might be moving away from the service, but at least you've got the time to be able to really explore what that option is before it explodes, before the ticking time bomb explodes and you have to leave. | | | |
|  | Jay- So for them having to change their whole psyche of society and within the job to admit and accept that help I think is hard. Services that focus on that I think are really important. So there are some career management programs through Fortem and the Ambulance has one as well. So if you do want to transition out, not just into retirement, what does that look like for you? I think that's giving options to help people not feel stuck in a career that's damaging or hurting them as well. | | | |
|  | Tabitha- Barriers would be stigma, fear about career progression, access, available appropriate services. Enablers are initiatives by the Department to provide supports, to make them available, to reduce stigma. Culture change as well. | | | |
|  | Ruth: Okay. I think it varies, depending on the stigma that’s attached, their identity within their town or community, whether they want to put up their hand or not. It’s really important for their organisations to be offering that support so that they don’t have to self-investigate where they might be able to get some assistance. Even though we spoke about EAP, at least that’s a good start that organisations give it. But it’s not the be and end (sic) to assisting them. | | | |
|  | Marissa: Yeah. I would agree with [Ruth] and I think there’s a lot of variability. I feel like I’m seeing reductions in stigma and more self-referral and people would be more willing to kind of say, I think there’s something going that I could benefit from some support. But often there are a lot of caveats and there’s a lot of need for sort of a tailored service. We find a lot of people are much more likely to identify with the idea of burnout rather than depression for example. Sometimes clinically that might be more appropriate. But that sense of, my role is a big driver in what’s going on. | | | |
| **Subtheme 2: *Recommendations and strategies for improving mental health*** | | |  | |
| Empowerment | Suzette- In the past, I did have this mentality that I’m going in and I’m going to fix it for them, but it doesn’t work that way. Because after the one or two weeks that I’m there, then they’re left to their own devices and unless I’m able to connect them to their own services then they’re not going to get the long-term help that they need. It’s not about going in and being the hero, it’s about going in and empowering the community to be able to meet their own needs during the disaster. | | | |
|  | Suzette- So, yeah, it’s been an eye-opener for me and a different way of practising, but I think it’s what’s best for the community rather than what’s best for me, for my recognition or whatever. It’s not about me, it’s about the people. So shifting that tact to being more of a social worker that can refer them on to their own services and make requests to government to say that they don’t have enough services therefore we need to build on their services because this is a disaster-prone area. Then it’s what we’re hoping to do with [unclear] and other places like that that suffer regularly with floods, so that they can grow and provide for their own people. It’s a challenge. It needs money. | | | |
|  | Marissa: Yeah. Look, with my experience, I haven’t really been involved in any direct group level interventions. But the things I’ve heard about that seem to have a lot of benefit is things that target that level of isolation and community support. So, like Ruth was saying, not just maybe seeing a psychologist or a psychiatrist, but also people who are involved with your church or spiritual community. People who are – maybe with lived experience. I feel like a lot of clients seem to find those spaces extremely empowering and supportive and they might not be kind of treating what’s going on, but really remove the disability and functional impairments that can be associated with that by supporting them with suicidal ideation or sense of loss that can happen. Especially if people have lost their roles due to injuries or illness or trauma. | | | |
|  | Emma- There are a lot of systems-level issues that are perpetuating these mental health issues that we are not looking at. So a lot of these we’ve touched on today - like there are things that the agencies and the organisations need to do to prevent - like if we empowered all of our people leaders with the knowledge and skills, and confidence and capability, to look after their people that would make a huge difference, right? | | | |
| Individual strategies to mitigate mental health related issues | Suzette- So for retired members, there is a great need of added support. Because if they haven’t had the support earlier on, then the compounded trauma of - whether it’s moral injury, whether it’s PTSD, compassion fatigue, it’s just going to stay with them and fester. Unfortunately, when they start looking at what the organisation is not doing for them as what it used to do in the past, they get more disgruntled, more depressed, more isolated, and everything is just compounded. So, yes. I feel that for retired members - and now I’m journeying with a few retired members that just feel like they’ve been abandoned by the organisation. That’s what was spoken about before. So I’m really conscious of and targeting some of our retired members. Particularly because in St John if you’ve done extraordinary stuff you are acknowledged in the Order. So they can become members of the Order. We have got at the moment really highly decorated members of the Order who all of the sudden - you know, they’re a dame in the Order and now nobody is talking to them. Nobody is keeping them connected and they feel that all the supports have gone. So, yeah. I’ve got them on my list [laughs].. | | | |
|  | Maise: I think one of the things - I don’t actually like to use the word preventative. I like to use the word fitness, resilience fitness. Because it implies that you’re adding strength as opposed to trying to fend off something negative or trying to band-aid or wrap a wound, whether it be physical, or emotional, or mental. Whereas if you can build up your fitness skills - just as you build up your training to be a good responder, so to - you need to build up your fitness skills to deal with some of this stuff. We also need to teach our organisations and our communities about patience. A lot of this stuff isn’t relevant until it’s relevant and we can have all of this training and we can put all of these things into place, but it’s not relevant until it’s relevant, if that makes sense? Yep. I don’t know what I need until I need it, and my needs are going to be different from each of your needs. So that’s something. We all know with mental health, it’s not a quick fix. | | | |
|  | Lucas- Resilience traits, I don’t think there’s a resilient trait - I really don’t know what that terminology is in regards to, I’ve got something different to what someone else might have. A good peer support system I’ve seen work really well, like an effective peer support group that the staff actually trust, which would often be a referral then to an EAP service. Like Karie said, the debrief, if done effectively. As in a caring debrief I should say, if done effectively where they actually genuinely care for the welfare of the person, not based on what procedures were done. If it’s done on a caring aspect, it’s really quite good and effective. | | | |
|  | Jay- Yeah, you're not always going to get what you want, but how can we help you? Even though we're going to employ you, how can we help you better? What does that look like for you? If we can't, how can we personalise it as much as we can to help you? I think that individualised treatment, whilst it might seem like a lot of investment, the introduction - those that have had amazing introductions stay and they build resilience and they feel confident in themselves and their workplace to be able to do their job. | | | |
|  | Ruth- Healthy lifestyle program which Marissa spoke about too, but about eating well, having little competitions, morning teas and all that sort of stuff. But also, are you fit. We had a program, are you fit for work. Have you had enough sleep? Have you had a break from whatever? Have you not been drinking alcohol? Have you done some exercise? How do you feel today? Are you ready for work? | | | |
|  | Jay- People after that always say they feel a lot better. So I think that mindfulness practice is good and a certain degree - not diet - people say - I feel like people are happier in regional and remote areas and they're like, yeah, I can't control much but I've started to eat better and there's a sense of pride that comes with, I've been eating better and I've been exercising a bit more and it's correlating with each other as well. So again, not massive strategies, but just little self-improvement strategies seems to help people I think | | | |
|  | Tate- Well, this may sound silly, but introduction of gyms on the stations. A lot of the time it's jump on the bike or on the treadmill or go pump some iron, that sort of thing. When you can't get away because a lot of the time - and it's changed a bit since I was in the smallest stations, where there used to be two of us, now there's five or six, so you do get the opportunity to go, I want go for a run, I want to go jump on my bike, or I want to go - one of my saviours has been swimming. No voices, no phones, no nothing, just you in the water. Does that answer that one? | | | |
|  | Jay- So it's a bit of personal resilience that's built up and strategies. People that have gone to psychologists before. I know at uni we're like, if you feel like things are stressed or you get a bit anxious or anything like that, it's not going to hurt to go and see someone. Before you join an ambulance service, maybe have a look at that and just go, I've got this. I'm going into this role. What are some coping strategies or what can I do in that space and starting that conversation earlier pre-emptively I think is helpful. | | | |
| Support & Interventions | Simon- I believe that more so in our country here that they don’t have critical incident and debriefs on - well, it’s up to whoever is running the police station at the time. If it’s an inspector and they don’t think that this incident deserves a critical incident debrief, then they won't do it. They’ll just say, ring the chaplain or ring the employee assistance program or whatever. Whereas, if it was an oversight board, committee, whatever, that says every critical incident needs to be dealt with this way, every - the ambulance, I think, do the better job of their critical incidents. I don’t know whether that is because doctor - why can't I remember his name, their commissioner. | | | |
|  | Suzette- I’m wondering whether inter-agency supports would be helpful. Because I know that we all have our own structures of EAP, Peer Support, and chaplaincy, but I’m wondering - if you don’t have chaplains for SES in say this area but you have one for St John, I’m wondering whether we could be sharing the logistics of where are our chaplains or where are our people inter-agency wise. So that if there’s not a chaplain for SES in the [Gunning] area, then they know that I’m here and that they can turn to me if they need to. So I’m wondering if something like that would actually be helpful? | | | |
|  | Ruth- Then the other thing was to really make people – some of the commanders – genuine about their efforts to get people back to work. Whether it was two hours a week. Whether it was two hours a week. Whether it’s just they came in and have coffee. Whether it then went to two hours for two days or whatever. That there was a journey that they had to go on with the officer to get them back to work and look at alternate duties. Because if they’re off you’ve lost that person – that unit anyway, so you may as well try and put a bit of effort to get them – and if they have to be working in the radio room for example instead of out on the truck, well you’ve still got some work being done by them. But they’re also then not sitting at home feeling depressed and not with their support team. So that worked really well as well. | | | |
|  | Maise: I think rural communities have an in-built resilience probably greater than people who live in towns and cities. They are used to doing things on the fly with a piece of baling twine, with two kids, one under each arm and multiskilling and making the dollar go as far as it can go. Getting up on those tough days and still getting on with the job because you have to. There is no choice. I think it is our greatest strength and our greatest asset. When we turn around and we see a neighbour struggling, we know what it feels like to be that struggling neighbour and so we don’t think twice about going over and giving them a hand. It is actually, for all of our challenges and for all of the things that we don’t do well, for all of the things that we don’t have access to in our rural and remote communities, what we do have is an innate sense of character and resilience and that stuff can’t be bought anywhere. That’s our greatest asset. | | | |
|  | Suzette: I think as far as families are concerned in rural areas, I think that the family connections are less nuclear than what city responders have. I think that the family connections - you know, it takes a community to raise a child. In rural settings, there is more of a community understanding and a community coming together rather than in cities. So I think that is going to be a major difference between first responders in the city area and in a rural setting. That there are going to be more people. Because I know from divisions in the rural settings, it is a family being involved in that group, in say Saint John [unclear] there’s that kind of family attitude. Or the [Kuma] group, they are a group. They get together, they celebrate together, they grow together. So I think that that is stronger in the rural settings than it is in the cities, where they just come and go. | | | |
